# An open-label, multicohort Phase Ib trial of pembrolizumab (MK-3475) for advanced hematologic malignancies: KEYNOTE-013

**DOI:** 10.1186/2051-1426-3-S2-P169

**Published:** 2015-11-04

**Authors:** Vincent Ribrag, Phillippe Armand, John Kuruvilla, Jean-Marie Michot, Craig H Moskowitz, Patricia Marinello, Ellen Snyder, Arun Balakumaran, Margaret A Shipp, Pier Luigi Zinzani

**Affiliations:** 1 Gustave Roussy Cancer Campus, Villejuif, France; 2 Dana-Farber Cancer Institute, Boston, MA, USA; 3 Princess Margaret Cancer Centre, Toronto, ON, Canada; 4 Memorial Sloan Kettering Cancer Center, New York, NY, USA; 5 Merck & Co., Inc., Kenilworth, NJ, USA; 6 University of Bologna, Bologna, Italy

## Background

Tumors, including hematologic malignancies, can use the PD-1 pathway to evade immune surveillance. Pembrolizumab, a highly selective, humanized monoclonal antibody that blocks interaction of PD-1 with its ligands PD-L1 and PD-L2, has demonstrated robust antitumor activity and a manageable toxicity profile in advanced solid tumors. In earlier data, pembrolizumab demonstrated efficacy in Hodgkin lymphoma (HL)[1]. KEYNOTE-013 (ClinicalTrials.gov, NCT01953692) is a multicenter, nonrandomized, open-label, multicohort Phase Ib trial designed to assess safety and efficacy of pembrolizumab in patients with hematologic malignancies.

## Methods

Cohorts include patients with intermediate-1, intermediate-2, or high-risk myelodysplastic syndrome (MDS) who failed ≥4 cycles of prior treatment with a hypomethylating agent; relapsed/refractory (R/R), multiple myeloma (MM) who failed ≥2 lines of prior therapy, including a proteasome inhibitor and IMiD; or R/R non-Hodgkin lymphoma (NHL): primary mediastinal large B cell lymphoma (MLBCL), follicular lymphoma (FL), diffuse large B cell lymphoma (DLBCL), or any other PD-L1-positive NHL that failed, was ineligible for, or refused a stem cell transplant and R/R HL (not presented here). Key eligibility criteria: age ≥18 years; ECOG PS 0/1; measurable disease; adequate hematologic, renal, and hepatic function. Key exclusion criteria: immunosuppressive disorder, active autoimmune disease, active pneumonitis, prior anti-PD-1/anti-PD-L1 therapy, active CNS involvement, and prior allogeneic hematopoietic stem cell transplantation within 5 years). Pembrolizumab is given at 200 mg Q3W in patients enrolled under the last amendment. Treatment continues until disease progression or intolerable toxicity; clinically stable patients may continue treatment beyond radiographic or hematologic (MDS or MM) evidence of progression until confirmed in following assessment. The primary end point is objective response rate (ORR). Secondary objectives include duration of response, progression-free survival, overall survival, and association between PD-L1 expression and response. Target enrollment for DLBCL and FL cohorts is ~50 patients.

## Trial registration

ClinicalTrials.gov identifier NCT01953692.
